# Comparison of different detection methods for *Ascaris suum* infection on Austrian swine farms

**DOI:** 10.1186/s40813-021-00236-9

**Published:** 2021-10-19

**Authors:** Anja Joachim, Christian Winkler, Ursula Ruczizka, Andrea Ladinig, Michaela Koch, Alexander Tichy, Lukas Schwarz

**Affiliations:** 1 Institute of Parasitology, Department of Pathobiology, University of Veterinary Medicine Vienna, Veterinaerplatz 1, 1210 Vienna, Austria; 2 University Clinic for Swine, Department for Farm Animals and Veterinary Public Health, University of Veterinary Medicine Vienna, Veterinaerplatz 1, 1210 Vienna, Austria; 3 Bioinformatics and Biostatistics Platform, Department of Biomedical Sciences, University of Veterinary Medicine Vienna, Veterinaerplatz 1, 1210 Vienna, Austria

**Keywords:** Pig, Roundworm, Serology, Screening, SERASCA®, Milk spots, Copromicroscopy

## Abstract

**Background:**

*Ascaris suum*, the large roundworm of pigs, is one of the economically most important pig parasites worldwide. In Austria it is commonly diagnosed by monitoring livers for milk spots at the slaughterhouse and intravital diagnosis (flotation for detection of fecal egg shedding). Recently, serological diagnosis based on the detection of specific antibodies with an ELISA (SERASCA®) with high sensitivity has been developed. To introduce and evaluate serology for *A. suum* screening in Austrian pigs, blood (for serology) (n = 177) and feces (for copromicroscopy) (n = 177) were taken from randomly selected slaughter pig batches from 18 farms at a slaughterhouse in Lower Austria. In addition, livers presented at slaughter (n = 844; max. 70/farm) were evaluated for milk spots.

**Results:**

Overall, 19% of the livers were milk spot-positive (22% of those with complete diagnostic evaluations). Thirteen percent of the fecal samples contained *A. suum* eggs, while 69% of the blood samples were serologically positive. Despite we did not determine the sensitivity of the ELISA specifically, results ouf our study confirmed the high sensitivity of the ELISA, which was claimed by the manufacturer prior to our work (sensitivity: liver assessment: 23.5–27.0%; copromicroscopy: 8.5–9.0%; ELISA: 99.5%), and a high percentage of *A. suum* infections that remained undetected by standard liver assessment.

**Conclusions:**

This suggests that the current method of roundworm diagnostics is insufficient and antibody detection at the end of the fattening period should be established as the standard procedure.

## Background

Infections with the large roundworm, *Ascaris suum,* are frequent in pigs worldwide with often high prevalences [1–8]. After ingestion of infectious eggs, larvae will hatch in the intestine and migrate to the liver and to the lung from where they will return to the digestive tract to develop to fertile adults [9]. Infections with high numbers of larvae can cause acute pneumonia [10]; however, in most cases uptake of a low to moderate number of eggs leads to chronic infection with establishment of adults in the small intestine followed by the continuous production of large numbers of eggs which, once embryonated, will lead to continuous reinfection [4]. Migrating larvae cause alterations of liver tissue which demarcate as distinct granulomatous to fibrous alterations, so called milk spots, which lead to the condemnation of affected livers upon slaughter. Reinfection leads to reduced worm burden as immunity develops [3, 9], and towards the end of the fattening period egg excretion can be low or absent despite continuous ingestion of eggs which leads to milk spot development that can be increased in reinfections as a sign of activated immune responses in the liver [4, 11]. Apart from liver condemnation, *A. suum* infections can also compromise weight gain, feed conversion efficacy as well as meat quality [12–18].

Immunity to *A. suum* is of a mixed cellular and humoral (Th_2_) type [19, 20]. Although mechanisms of protective immunity against *A. suum* are related to intestinal compartments [20, 21], serum antibodies are induced upon infection and are related to adverse effects on the developing worms [22]. In addition, they can also be used for diagnostic purposes. The usefulness of serodiagnosis using *A. suum* hemoglobin as antigen to determine infections in fatteners [18] and nursing piglets [23] was demonstrated earlier. Subsequently, a commercial serological test was developed for this purpose in an ELISA format (SERASCA®; www.serasca.com) for the detection of specific anti-*A. suum* IgG [22].

The present study compared *Ascaris* egg excretion, liver lesions and antibody detection in slaughter pigs to determine infection rates in the examined population and to evaluate the suitability of the different assays for the detection of *A. suum* infection on fattening farms in Austria.

## Results

**Fig. 1:**
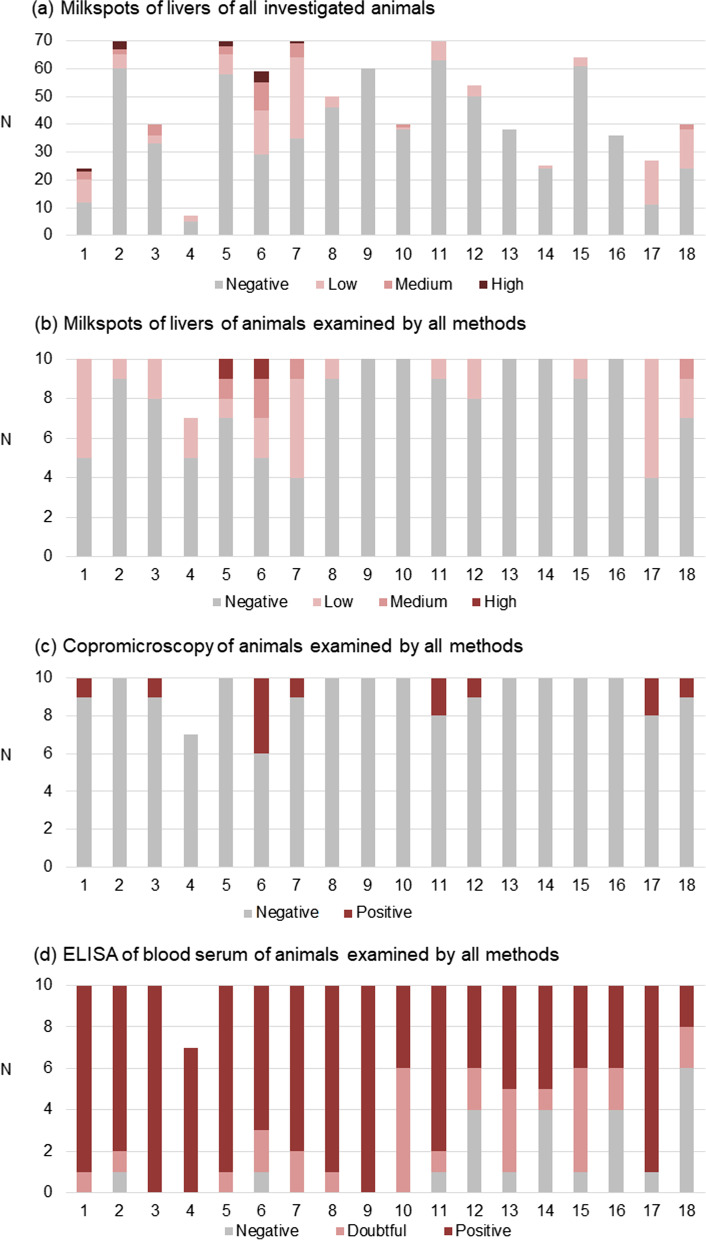
Results of **a** liver inspection of all investigated animals (n = 844 livers), **b** liver inspection of animals that were also screened by copromicroscopy and ELISA (n = 177 animals). Each bar indicates the corresponding farm of the examined animals. Positive livers showed different grades of milk spots: low (< 5 visible milk spots on the surface), medium (5–20 milk spots), high (> 20 milk spots). **c** Copromicroscopical analysis of the same animals as in (**b**) by modified McMaster egg counting. Only qualitative results are shown. **d** ELISA for anti-*Ascaris suum* serum antibody detection from the same animals as in (**b**) and (**c**). ODR values > 0.6 were considered as positive, ODR < 0.4 as negative, and values 0.4–0.6 as doubtful

Overall, 844 livers from 18 farms were evaluated for milk spots. Milk spot positive livers were detected on 15 farms (83.3%) and in 19.1% of the samples. On average each farm had 20% (0–59.3%) positive livers (Fig. 1a).

Of the animals (n = 177) for which complete examinations of liver, feces and serum was available, 22% were positive for milk spots (82.1% of these had low and 12.8% medium grades), and 7.4% excreted *Ascaris* eggs (Table 1; Fig. 1b). The mean excretion rate was 2396 eggs per gram of feces (epg) (standard deviation: 2331), the median 1600 epg (Tables 1 and 2; Fig. 1c). Other endoparasites were not detected by copromicroscopy. Antibodies were detected on all farms and 13.6% of the samples were negative, while 68.9% were positive and 17.5% were doubtful (Table 1; Fig. 1d).

**Table 1:** Results of liver inspection, copromicroscopy and serology of 177 animals from 18 different farms

Parameter	N positive farms (%)	N positive samples (%)
Total number	18 farms	177 livers
*Liver inspection*		
Milk spots total	13 (72.2)	39 (22.0)
Low grade (< 5)	13 (72.2)	32 (18.1)
Medium grade (5–20)	4 (22.2)	5 (2.8)
High grade (> 20)	2 (11.1)	2 (1.1)
*Fecal examination*		
Eggs in feces (McMaster positive)	8 (44.4)	13 (7.4)
*Serology (SERASCA®-ELISA)*		
Positive	18 (100)	122 (68.9)
Doubtful	0 (0)	31 (17.5)

**Table 2:**
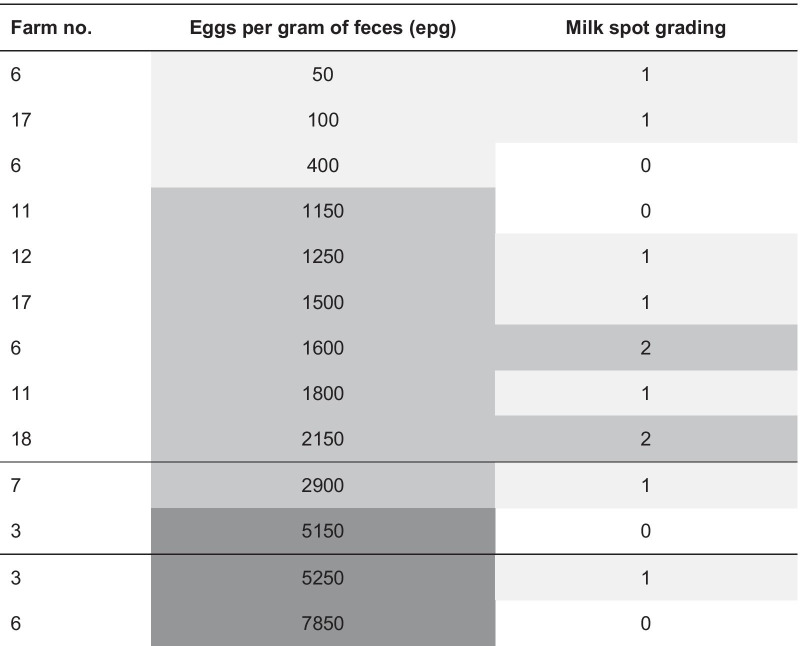
Results of liver inspection in individual animals positive for *A. suum* in fecal examination (N = 177 samples in total, 13 positive in fecal examination)

The farm number indicates the origin farm of the corresponding animal. Grey shades indicate grading of eggs per gram of feces (low: ≤ 1000 epg, medium: 1000–3000 epg, high: > 3000 epg) and milk spots (0 = no milk spots; 1 = low: < 5, 2 = medium: 5–20, 3 = high: > 20)

When the different diagnostic tests were compared using ELISA as the gold standard, the sensitivity of liver inspection was 23.5–27.0% and its specificity 87.5–89.1% with positive predictive values of 84.6–92.3%. The agreement was low and only significant when the doubtful ELISA results were considered as negative. The sensitivity of the fecal examination was < 10% with a high specificity (96.4–100%) and a low and insignificant agreement in all comparisons (Table 3).

**Table 3:** Comparison of different diagnostic tests (statistical analysis) taking the results of the serological examination (ELISA) as the gold standard

Reference	Liver inspection^a^	Fecal examination^a^
ELISA (positive + doubtful results considered as positive)	Sens: 23.5	Sens: 8.5
	Spec: 87.5	Spec: 100
	PPV: 92.3	PPV: 100
	κ: 0.037	κ: 0.0025
	P: 0.225	P: 0.138
ELISA (only positive results considered as positive)	Sens: 27.0	Sens: 9.0
	Spec: 89.1	Spec: 96.4
	PPV: 84.6	PPV: 91.0
	κ: 0.114	κ: 0.035
	P: 0.017	P: 0.204
ELISA (doubtful results excluded)	Sens: 27.0	Sens: 9.0
	Spec: 87.5	Spec: 100
	PPV: 91.7	PPV: 100
	κ: 0.131	κ: 0.032
	P: 0.060	P: 0.126

^a^Sens: sensitivity [%]; Spec: specificity [%]; PPV: positive predictive value [%]. P: Probability associated with κ

## Discussion

To evaluate the usefulness of serology for the detection of *A. suum* infections in Austrian slaughter pigs of conventional indoor farming systems, a comparison between three different methods, liver inspection, copromicroscopical examination and detection of antibodies by serology was undertaken on non-randomized samples from an Austrian slaughterhouse. Since sampling was not stratified for the clustering of *A. suum* on farms or pens, the data do not reflect prevalence rates on Austrian swine farms but were only used to evaluate the usefulness of the different assays. For the same reason, the serological testing was conducted in “unicates” as recommended by the manufacturer, not in duplicates or triplicates to evaluate the variability of the test, as previous work already determined the specificity and sensitivity of the SERASCA® ELISA [22].

Until recently, the routine diagnosis of *Ascaris suum* infections on swine farms was limited to copromicroscopy and, where provided, feedback from the slaughterhouse regarding liver inspection results. Liver condemnation is currently sanctioned by some slaughterhouses in some countries which can cause considerable losses (€ 2.50/liver; [24]). In addition, reduced feed conversion, a reduced lean meat content, and an increased susceptibility to respiratory pathogens and corresponding expenditures for treatment add to the costs of roundworm infection which amount to € 5–8/pig [12, 13, 16–18, 25–27]. However, in some of these studies it was not possible to demonstrate an economic effect of treatment. This may have been due to the fact that in such studies the corresponding diagnostic method had a poor sensitivity and consequently resulted in misleading conclusions and/or lack of measurable effects. Consequently, routine treatment is frequently advised and administered to fattening pigs after weaning under the assumption that *A. suum* is present on a farm. However, in cases where roundworm infections do not impair animal health or production economy, treatment is not only redundant and economically irrational, it also increases chemical residues in pork and, via manure, in the environment, both undesirable side effects of anthelmintic treatment that must be avoided.

On the other hand, it has to be acknowledged that on the farm level, both meat inspection reports and fecal examination are of poor sensitivity since only a limited number of samples are examined at a given time point and may not accurately reflect the infection status of a herd. In addition, these diagnostic analyses only return information about the presence of the parasite on a farm after the onset of egg excretion (for copromicroscopy) or after subsequent environmental contamination with infectious worm eggs (for liver inspection). Results from slaughterhouse inspections are also not always available in sufficient detail and may not return sufficiently accurate results, and this is also a limiting factor for copromicroscopy [28] when only a low number of samples are examined *e.g.* for economic reasons. On the other hand, serological examination has a higher sensitivity than the other two tests and can consequently be applied on a small number of samples per farm for the indirect detection of *A. suum* [22]. This limits the costs for testing and also the workload for taking samples. Blood samples do not have to be taken specifically for the detection of anti-*A. suum* antibodies but can be used to evaluate the overall health status of the herd regarding a number of swine pathogens, including, *e.g.*, sarcoptic mange, APP, PRRSV, or influenza A virus. This would not only decrease costs but also manipulation and handling stress of animals, and thus contribute to animal wellbeing.

As for other indirect methods, serological testing for *A. suum* is of no value when the presence of infections on a farm has already been determined otherwise, but it will be valuable on farms with a low infection pressure as infections can be determined in the prepatent period (although not long before egg excretion can be expected [22]) and also in animals that were infected but do not harbor adult worms, either due to successful treatment shortly before testing, or because of the onset of immunity and subsequent abortion of nematode development after infection. This warrants considerations about the aims of serological testing and the optimal time point for sampling. The SERASCA® assay clearly aims at determining the presence of *A. suum* in a herd with long-term exposure and not in an individual [22]. Thus, detection of positive samples implies that measures for its control must be considered, since even single patently infected animals can quickly shed eggs in high numbers and contaminate the environment with infectious stages [1, 3, 4]. Repeatedly negative test results, however, imply that control of *A. suum* on the tested farm is sufficient. To increase the chance of detecting infections, the time point of highest antibody titers after infection needs to be considered. Seroconversion was observed from 6 weeks after experimental infection with *A. suum*, culminating 2 weeks later. Another study found out, that anti-*A. suum* antibodies in experimentally infected piglets were detected for 90–100 days after infection peaking between the 20th and 40th day after infection [29]. Considering that *A. suum* is of highest significance during fattening, and that infection most frequently takes place when animals are relocated to the fattening unit, serology should be undertaken in animals from at least 18 weeks of age or more than 60 kg of life weight [22]. This scenario also reflects the epidemiological situation on Austrian conventional indoor swine farming systems. Taking into account that in European countries different farming systems are implemented in swine production (e.g., outdoor farming with huts; organic pig farming; deep straw bedding), the optimal sampling time point may be at an earlier time point or at a body weight less than 60 kg.

Considering the life cycle of *A. suum* and the immunological responses induced by infection, the varying correlations between the different detection methods were to be expected. The correlation between the results of serology and liver examination in the present work and in a previous study [18] indicated that the extent of liver damage can be estimated by serology (indicating that migrating larvae induce detectable antibodies, especially after reinfection). A higher sensitivity of the ELISA can be assumed since milk spots appear quickly (about 3 days) after infection but disappear again within 2 to 3 weeks after experimental infection [9, 30] whereas antibodies can be detected for several weeks [18, 22]. A correlation was also shown for lung lesions and increased antibody titers in a previous study [18]. In contrast to that, results of copromicroscopy were not correlated with serology in the present study and a previous one in humans with *Ascaris* infections [28]. This could be due to a limited antibody response elicited by intestinal, reproducing adult stages. After primary experimental infection with *A. suum*, antibodies against *Ascaris* hemoglobin could be detected in fatteners but not in piglets [23]. By contrast, an ELISA based on whole *Ascaris* antigen derived from larval stages in the lung of experimental piglets could clearly show seroconversion from the 4th week after infection [22, 23]. This indicates that the SERASCA® test provides valuable data for the screening of fatteners, but not of suckling piglets, although a single study does not permit a definitive decision on this point.

The applicability and usefulness of the *Ascaris-*ELISA will have to be further evaluated for different settings, not only for prevalence screening as in previous studies [18, 28] but also for treatment efficacy and, should this be attempted, measures for complete eradication of large roundworms from a farm. In the latter case, some cross-reactivity with the porcine whipworm *Trichuris suis*, should be considered [22].

## Conclusions

As demonstrated previously, the SERASCA® ELISA is a sensitive indirect test for the detection of *A. suum* infection in swine herds, and should be considered as a diagnostic tool in herds with a low infection level (e.g. to facilitate the decision of targeted treatment of weaners at the start of fattening) that cannot readily be detected by conventional methods, and for efficacy screening of intervention strategies. Liver inspection, should, however, not be neglected as a diagnostic tool, since it targets a different age group and provides relevant direct information on the economic impact of *A. suum* on a fattening farm at a given time point (most likely not more than 3 weeks prior to slaughter). The present work has shown that *A. suum* was highly prevalent on the investigated Austrian farms and serology was not the diagnostic method of choice for most of them as infection rates were high. Moreover, a blood test at the end of the fattening period can be a safe tool to evaluate if an infection occurred at an earlier stage in the pigs’ life. However, should fattening farms (or closed farms) decide to attempt to significantly reduce the infection level with *A. suum,* a more sensitive detection method will be required and a switch from liver lesion scoring to the ELISA would be recommended to accurately react with accompanying reduction measures.

## Materials and methods

### Samples

Samples were all taken at a slaughterhouse in Lower Austria on five consecutive days in April/May 2019. Blood was sampled from 18 conventional indoor farms (10 pigs per farm) during exsanguination using serum tubes without anticoagulants (Serum Primavette®, KABE LABORTECHNIK GmbH, Nümbrecht-Elsenroth, Germany) and fecal samples were collected from the corresponding intestines (rectum). From one farm (no. 4) only seven livers, blood and fecal samples could be obtained due to technical reasons, as three individual intestines could not be assigned to the corresponding serum and liver samples. Therefore, these three samples were excluded from the data set.

### Milk spot grading

Livers of the sampled animals and additionally of pigs belonging to the same farm [max 70 livers/farm] were inspected and scored as negative, low grade [< 5 visible milk spots on the surface], medium grade [5–20 milk spots] or high grade [> 20 milk spots]. For determination of sensitivity and specificity ≥ 1 milk spot was counted as a positive result. To obtain more data on the liver condition during meat inspection, animals from each slaughter batch were evaluated completely while blood and fecal samples were taken from a subset only.

### Fecal examination

Fecal samples [n = 177] were examined copromicroscopically after concentration by sedimentation in water and flotation of the sediment in saturated sugar solution (specific gravity: 1.3 g/cm^3^) by centrifugation (690×*g*, 8 min). The flotate was transferred to a glass slide, covered with a cover slip and examined under 100 × magnification. In positive samples, egg per gram of feces (epg) were determined using a modified McMaster technique with concentrated sugar solution. Four gram of feces were mixed with 60 ml of flotation solution and two counting chambers (150 µl each) were filled and counted. Egg concentrations were calculated as follows:

1 $$\begin{aligned} {\mathrm{epg}} & = \frac{{{\mathrm{Eggs\,counted\,in\,two\,counting\,chambers}}\,*\,{\mathrm{suspension\,volume}}\,\left[ {{\mathrm{ml}}} \right]}}{{{\mathrm{Amount\,of\,feces}}\,\left[ {\mathrm{g}} \right]\;*\;{\mathrm{volume\,of\,2\,counting\,chambers}}\,~\left[ {{\mathrm{ml}}} \right]}} \\ {\mathrm{i.e}}.\quad {\mathrm{epg}} & = \frac{{{\mathrm{Eggs\,counted}}\,\,{\mathrm{*}}60}}{{4\,*\,0.3}} \\ {\mathrm{or}}\quad \quad {\mathrm{epg}} & = {\mathrm{Eggs\,counted}}\,*\,50 \\ \end{aligned}$$

### Serology

Blood samples were centrifuged (10 min, 805×*g* at room temperature) and serum was removed and stored at − 20 °C until analysis. All sera (n = 177) were analyzed with the SERASCA® ELISA (Boehringer Ingelheim, Vienna, Austria) according to the manufacturer’s instructions. Samples were processed in an automated ELISA processing system (Dynex DS2®, Dynex Technologies Inc., Chantilly, VA, USA), optical density (OD) was measured at 450 nm and the optical density ratio (ODR) was calculated as follows:

2 $${\mathrm{ODR}} = \frac{{({\mathrm{OD}}_{{{\mathrm{sample}}}} - {\mathrm{OD}}_{{\mathrm{negative\,control}}} )}}{{({\mathrm{OD}}_{{\mathrm{positive\,control}}} - {\mathrm{OD}}_{{\mathrm{negative\,control}}} )}}$$

All controls were run in duplicates, the serum samples as unicates. The test was validated for running test samples as unicates, as specified in the manufacturer’s instructions. The test was considered valid when (a) mean OD_positive control_ ≥ 0.8, (b) OD_negative control_ < 0.5 and (c) mean OD_positive control 1_ – OD_positive control 2_ ≤ mean OD_positive controls_/4. ODR values > 0.6 were considered as positive, values < 0.4 as negative. Values of 0.4–0.6 were questionable.

### Statistical evaluation

Statistical analyses were carried out using IBM SPSS v24 (IBM, Armonk, N.Y, USA). Differences in frequency distributions were calculated using chi square tests. The agreement was calculated using Cohen’s Kappa (κ). κ-values were interpreted according to Viera and Garrett [31]. Only McMaster positive fecal samples were used for statistical analyses. For determination of sensitivity, specificity and the positive predictive value (PPV), crosstabulations were calculated. For all statistical analyses a *p*-value below 5% (*p* < 0.05) was seen as significant.

## Data Availability

All data and materials of this study are contained in this manuscript.

## Abbreviations

APP: *Actinobacillus pleuropneumoniae*
°C: Degree of Celsius
€: Euro
Epg: Eggs per gram of faeces
ELISA: Enzyme linked immunosorbent assay
g/cm^3^: Gram per cubic centimeter
κ: Kappa
kg: Kilogram
ml: Millilitre
µl: Microlitre
n: Number
OD: Optical density
ODR: Optical density ratio
P: Probability
PPV: Positive predictive value
PRRSV: Porcine reproductive and respiratory syndrome virus
Sens.: Sensitivity
Spec.: Specificity
